# Pass-through with volatile exchange rates and inflation targeting

**DOI:** 10.1007/s10290-023-00502-8

**Published:** 2023-05-26

**Authors:** Annika Alexius, Mikaela Holmberg

**Affiliations:** Department of Economics, Stockholm University, 106 91 Stockholm, Sweden

**Keywords:** Exchange rates, Inflation, Pass-through, Bayesian time-varying parameter VAR, F41, E31

## Abstract

As central banks struggle against high inflation in the aftermath of the Covid-19 pandemic and the war in the Ukraine, it is essential to understand the open economy aspects of inflation determination. Using a Bayesian VAR with time-varying parameters and stochastic volatility, we analyze the behavior of pass-through across time and in relation to macroeconomic variables. Pass-through increases with the size of the volatility of the exchange rate and the level, variance and persistence of shocks to domestic prices, which is in line with theory. The persistence of exchange rate shocks is associated with higher pass-through only for observations with low inflation. Furthermore, the effect of inflation persistence on pass-through is much higher for exchange rate appreciations than for depreciations.

## Introduction

Following the Covid-19 pandemic and the Ukrainian war, inflation rates areprofoundly overshooting their targets in many countries. As differenteconomies are affected asymmetrically by shocks, large swings in nominalexchange rates are observed. These movements in turn affect inflation. The US dollar has appreciated 15% since 2021 and the US inflation rateslowed down before other countries. On the other hand, the effects onJapanese inflation of the recent 20% depreciation have been hithertobeen moderate. The British Pound depreciated by 36% 2007–2009, followed by inflation rates approaching 5%. The Japanese Yen fell by 44% between 2012 and 2014, which contributed to the highest Japaneseinflation rate in several decades. The 20% appreciation of the Swissfranc in 2015 was followed by several years of deflation. The pass-throughof nominal exchange rate changes to inflation appears to vary over time andbetween countries. Given the central importance of the inflation rate in themacroeconomy, studying the link between nominal exchange rates and inflationis essential.

This paper employs Bayesian vector autoregressive models with time varying parameters and stochastic volatility to estimate the pass-through of nominal exchange rates to prices using data on eight open economies. A measure of the time varying pass-through is collected and regressed on macroeconomic variables that may influence the size of the effect according to theory.

Following Campa and Goldberg (2005), a sizable literature analyzes the determinants of exchange rate pass-through. In models where price changes are costly, pass-through increases with the level, variance and persistence of inflation, and the persistence of exchange rate changes. Depreciations have larger effects than appreciations since prices are increased also in the absence of exchange rate movements. Several studies use cross country and/or split sample regressions to capture the relationships between pass-through and macroeconomic variables. Gagnon and Ihrig (2004), Devereux and Yetman (2010), and Choudhri and Hakura (2006) confirm Campa and Goldberg (2005) findings that low and stable inflation is associated with low pass-through. As discussed by Chou (2019), much more information on the behavior of pass-through can be extracted by allowing it to vary continuously over time rather than have one observation per country. The opposite approach is taken by Auer et al. (2021), who instead move to microdata and obtain an unusally sharp identification of the effect of exchange rate changes on domestic prices.

Ozkan and Erden (2015) define pass-through as the contemporaneous correlation between exchange rates and consumer prices within a Dynamic Conditional Correlation-Generalized Autoregressive Conditional Heteroscedasticity (DCC-GARCH). In a second step, panel regression using data for an extensive set of countries demonstrate that pass-through is positively related to the level and volatility of inflation and negatively related to exchange rate volatility. This measure of exchange rate pass-through is essentially zero after 1985 for industrialized countries. Chou (2019) estimates a time varying measure of exchange rate pass-through using quantile regression techniques. Consistent with the studies above, he finds that inflation volatility is positively associated with pass-through, while and exchange rate volatility is negatively associated. Higher exchange rate variability has different effects in different theoretical models and the empirical findings also differ between papers.

The TVP-VAR provides a time varying measure of pass-through based on a dynamic model, in contrast to the empirical specifications in Ozkan and Erden (2015) and Chou (2019) who also regress pass-through on a set of macroeconomic variables. Secondly, the framework allows us to study how pass-through is related to the persistent of shocks to exchange rates and prices in a time varying setting. Persistence is an important factor behind the degree of pass-through in models of with costly price changes or price stickiness. An and Wang (2012) and McCarthy (2007) study the relationship between pass-through and exchange rate persistence using time invariant correlations in cross country data. Here, a time varying measure of persistence is derived from the impulse response functions. More persistent inflation shocks is consistently associated with higher pass-through, while the persistence of exchange rate shocks only matters in some of the subsamples. Another novelty relative to previous studies is that we study pass-through for observations with low inflation or exchange rate appreciations. There are theoretical reasons to expect different associations between macroeconomic variables and pass-through for the extraordinarily low inflation rates observed since the financial crisis (Taylor, 2000; Devereux & Yetman, 2010). Possible mechanisms include increased macroeconomic volatility around the zero lower bound, more backward looking expectations in times of inflation underscoring, and higher persistence of shocks to inflation when the credibility of the inflation target is low. For the low inflation sample, the level of inflation is unrelated to pass-through and the variance of the exchange rate displays a positive association. Exchange rate appreciations could be passed on to domestic prices in a different manner than depreciations since prices are increased regularly (as long as inflation is positive). We find that exchange rate persistence has much larger effects on pass-through in case of appreciations, which is consistent with theoretical models where price changes are costly.

The remainder of the paper is organized as follows: Section 2 introduces the statistical model and Sect. 3 describes the data. The results are presented in Sect. 4, followed by conclusions in Sect. 5.

## Statistical model

We estimate a Bayesian VAR model with time-varying parameters and stochastic volatility. Two lags are included in the VARs and the first five years are used as training sample for OLS estimation of priors for the Bayesian models.

### The VAR with time-varying parameters

The empirical specification follows Primiceri (2005). Consider a multivariate model with *k* lags:

1 $$\begin{aligned} y_{t}=c_{t}+B_{1t}y_{t-1}+B_{2t}y_{t-2}+\cdots +B_{kt}y_{t-k}+u_{t},\qquad t=1,\ldots ,T \end{aligned}$$

where $y_{t}$ is a $n\times 1$ vector of endogenous variables, $c_{t}$ is a $n\times 1$ vector of time-varying constants, and $B_{it},i=1,\ldots ,k$ are $n\times n$ matrices containing time-varying coefficients for all lags. The error terms contained in the $n\times 1$ vector $u_{t}$ are heteroscedastic with a variance covariance matrix $\Omega$ that can be factored as:

2 $$\begin{aligned} \Omega _{t}=A_{t}^{-1}H_{t}\left( A_{t}^{-1}\right) ^{\prime },\qquad H_{t}=\Sigma _{t}\Sigma _{t}^{^{\prime }}, \end{aligned}$$

where $\Sigma _{t}$ is a time-varying matrix with error variances on the diagonal and $A_{t}$ is a time-varying lower triangular matrix with ones on the diagonal that contains the simultaneous relationships between the variables:

3 $$\begin{aligned} A_{t}=\left[ \begin{array}{cccc} 1 &{}\quad 0 &{}\quad ... &{}\quad 0 \\ a_{21,t} &{}\quad 1 &{}\quad ... &{}\quad ... \\ ... &{}\quad ... &{}\quad ... &{}\quad 0 \\ a_{n1,t} &{}\quad ... &{}\quad a_{nn-1,t} &{}\quad 1 \end{array} \right] \end{aligned}$$

4 $$\begin{aligned} \Sigma _{t}=\left[ \begin{array}{cccc} \sigma _{1,t} &{}\quad 0 &{}\quad ... &{}\quad 0 \\ 0 &{}\quad \sigma _{2,t} &{}\quad ... &{}\quad ... \\ ... &{}\quad ... &{}\quad ... &{}\quad 0 \\ 0 &{}\quad ... &{}\quad 0 &{}\quad \sigma _{n,t} \end{array} \right] \end{aligned}$$

Equations (1) and (2) imply that the VAR can be written as

5 $$\begin{aligned} y_{t}=c_{t}+B_{1t}y_{t-1}+B_{2t}y_{t-2}+\cdots +B_{kt}y_{t-k}+A_{t}^{-1}\Sigma _{t}\varepsilon _{t}, \end{aligned}$$

where the variance-covariance matrix of $\varepsilon _{t}$ is diagonal: $V\left( \varepsilon _{t}\right) =I_{n}$. The time-varying VAR can then compactly be expressed as

6 $$\begin{aligned} y_{t}=X_{t}{\textbf{B}}_{t}+A_{t}^{-1}\Sigma _{t}\varepsilon _{t}, \end{aligned}$$

7 $$\begin{aligned} X_{t}^{\prime }=I_{n}\otimes \left[ 1,y_{t-1}^{\prime },\ldots ,y_{t-k}^{\prime } \right] , \end{aligned}$$

where $\otimes$ denotes the Kronecker product and ${\textbf{B}}_{t}$ is a vector containing all right hand side coefficients of equation (5): $\textbf{ B}_{t}=vec\left( c_{t},B_{1t},\ldots ,B_{kt}\right)$. Equation (6) summarizes the processes for the time-varying coefficients. A standard assumption is that the dynamics of the time-varying coefficients (${\textbf{B}}_{t}$), simultaneous relationships ($A_{t}$), and the covariance matrix containing the standard deviations follow driftless random walks.

Denoting $a_{t}$ as a vector of non-zero, non-one elements in $A_{t}$ (stacked by rows), and $\sigma _{t}$ as the vector containing diagonal elements of $\Sigma _{t}$, the state equations of the model can be expressed as:

8 $$\begin{aligned} {\textbf{B}}_{t}={\textbf{B}}_{t-1}+\nu _{t} \end{aligned}$$

9 $$\begin{aligned} a_{t}=a_{t-1}+\xi _{t} \end{aligned}$$

10 $$\begin{aligned} \log \sigma _{t}=\log \sigma _{t-1}+\eta _{t} \end{aligned}$$

The innovations of the model follow a jointly normal distribution and have a joint variance covariance matrix *V*.

11 $$\begin{aligned} \left[ \begin{array}{c} \varepsilon _{t} \\ \nu _{t} \\ \xi _{t} \\ \eta _{t} \end{array} \right] \backsim N\left( 0,V\right) ,\qquad V=\left[ \begin{array}{cccc} I_{n} &{}\quad 0 &{}\quad 0 &{}\quad 0 \\ 0 &{}\quad Q &{}\quad 0 &{}\quad 0 \\ 0 &{}\quad 0 &{}\quad S &{}\quad 0 \\ 0 &{}\quad 0 &{}\quad 0 &{}\quad W \end{array} \right] \end{aligned}$$

The diagonal of *V* contains the hyperparameters of the model, which describe the tightness of the parameter distributions. $I_{n}$ is the variance matrix of $\varepsilon _{t}$ from Eq. (5). *Q*, *S* and *W* contain the variances of the innovations from Eqs. (8)–(10) and are defined as positive definite. *S* is assumed to be block diagonal, meaning that the simultaneous relationships between the variables can be estimated independently equation by equation.

### Priors

Choices of priors follow Primiceri (2005). The prior distributions of the initial states are estimated by OLS estimation of a fixed coefficient VAR on a training sample covering the first 5 years. The point estimates of $B_{0}$ and $A_{0}$ are set as means of the distributions for initial states of these parameters respectively (${\widehat{B}}_{OLS}$ and ${\widehat{A}}_{OLS}$ ). Conventionally, the variances are set to four times the variance of the OLS point estimates. The mean of the prior distribution for log $\sigma$ is set to the logarithm of the OLS point estimate (${\widehat{\sigma }}_{OLS}$), while the covariance matrix is set as an identity matrix $I_{n}.$

The hyperparameters *Q*, *S*, and *W* contain the variances of the state equation innovations, i.e. the tightness of the parameter distributions. We follow Cogley and Sargent (2001) and define *Q* and *S* as inverse Wishart distributions, and *W* as an inverse-Gamma distribution. Also in line with these authors, we restrict *W* to be diagonal. This partly departs from Primiceri, who also defines *W* as a scaled identity matrix, but uses inverse Wishart distributions for all hyperparameters. The priors can be summarized as follows:

12 $$\begin{aligned} \begin{array}{c} B_{0}\thicksim N\left( {\widehat{B}}_{OLS},~4Var\left( {\widehat{B}} _{OLS}\right) \right) \\ A_{0}\thicksim N\left( {\widehat{A}}_{OLS},~4Var\left( {\widehat{A}} _{OLS}\right) \right) \\ \log \sigma _{0}\thicksim N\left( {\widehat{\sigma }}_{OLS},~ 4_{I_{n}}\right) \\ Q\thicksim IW\left( k_{Q}^{2}\tau Var\left( {\widehat{B}}_{OLS}\right) ,\tau \right) \\ W\thicksim IG\left( \left( 1+\dim \left( W\right) \right) I_{n},(1+\dim \left( W\right) \right) \\ S_{b}\thicksim IW\left( k_{S}^{2}\left( 1+\dim \left( S_{b}\right) \right) Var\left( {\widehat{A}}_{bOLS}\right) ,(1+\dim \left( S_{b}\right) \right) \end{array} \end{aligned}$$

$S_{b}$ denotes the different blocks of *S*, $\tau$ is the number of observations in the training sample, and ${\widehat{A}}_{bOLS}$ refers to the respective blocks of the OLS estimate of simultaneous relationships (${\widehat{A}}_{OLS}$). The priors for initial states of the coefficients, simultaneous relations and standard deviations are assumed to be normally distributed. Together with the specified evolution of coefficients in (8)–(10), this implies that the whole sequence of parameters will be normally distributed.

The scaling matrices in *Q*, *W* and $S_{b}$ are constant fractions of their OLS estimates, where the OLS estimates are multiplied by corresponding degrees of freedom and scaling factors $k_{Q}$, $k_{W}$, and $k_{S}$. Degrees of freedom in each scaling matrix is set to size of training sample for *Q* and to one plus the dimension of the corresponding matrix for *W* and $S_{b}$. These numbers correspond to the minimum levels required to have proper priors, implying that the resulting priors only impose weak restrictions on the posteriors. Given the short period training sample, and hence the possibility of imprecise OLS estimates, it seems reasonable to avoid an approach that puts large weight on the priors. The small sample size of the training sample also motivates setting the parameters $k_{Q}=0.01$, $k_{S}=0.1$, and $k_{W}=0.01$ quite conservatively, i.e. in a manner that keeps priors rather diffuse. Primiceri (2005) uses the same calibration and calls these priors “ not flat but diffuse and uninformative”.

Bayesian methods are used to estimate the unobservable states and the hyperparameters of the joint variance covariance matrix *V*, i.e. the joint posterior of ($B^{T},$ $A^{T},$ $\sigma ^{T}$, *V*). Letting $\theta$ denote a vector of the unknown parameters ($\theta =\left( B^{T},A^{T},\sigma ^{T},V\right)$) and representing the data as $Y^{T}=\left( y_{1}^{^{\prime }},\ldots ,y_{T}^{^{\prime }}\right) ^{\prime }$, prior information about the distribution of these parameters is $\pi \left( \theta \right)$, while the likelihood function is $f\left( Y^{T}|\theta \right)$. Essentially, the posterior distribution $\pi \left( \theta |Y^{T}\right)$ is deduced by updating prior information about the parameters with information given by data.

Given stochastic volatility the state space model is non linear and numerical methods (Gibbs sampling) are used. To approximate the posterior distributions, 100 000 iterations of the Gibbs sampler is run, with an initial burn-in period of 30 000 observations to assure convergence to the ergodic distribution. To break autocorrelation of the draws, only every 10th draw is considered.

## Data

The data set covers eight countries: Australia, Canada, Japan, New Zealand, Norway, Sweden, Switzerland, and the United Kingdom. Quarterly data on nominal exchange rates, CPI, GDP, exports, and imports in constant prices are collected from OECD Main Economic Indicators. Data are transformed into logarithms and covers 1980Q1–2021Q3. Effective exchange rates and a trade weighted index of foreign prices are calculated for each country using BIS weights. Openness is measured as exports plus imports divided by GDP and output gaps are calculated using the Hodrick-Prescott filter with lambda equal to 1500.

## Results

The TVP-VARs contain three variables: Domestic prices, the effective nominal exchange rate, and trade weighted foreign prices. Two lags are included in the VARs and the first five years are used as training sample for OLS estimation of prior means for the Bayesian models. As originally proposed by Primiceri (2005), shocks are identified through a recursive structure on the contemporaneous effects with the ordering going from the top to the bottom variables. The ordering of variables is $\left[ P^{*}, P, E\right]$. Hence, exchange rate shocks are separated from shocks to domestic prices using the assumption that the exchange rates react to contemporaneous inflation shocks, while contemporaneous response of domestic prices to exchange rate shocks is zero.

The TVP-VARs produce one set of impulse response functions for each country in each time period. To study the evolution of pass-through over time and its relationship to macroeconomic variables, we collect the responses of domestic prices to a nominal exchange rate shock after four quarters. The TVP VAR also generates time varying volatilities as well as the half-life of shocks as measure of persistence.

### The development of pass-through over time

**Fig. 1:**
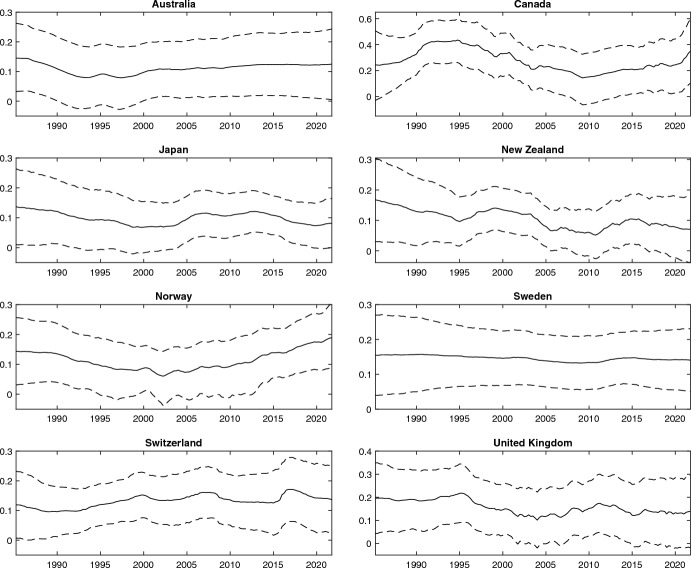
Exchange rate pass-through

There is general agreement that exchange rate pass-through has fallen since the 1980s (Gagnon & Ihrig, 2004). Whether the decline has continued to decline is a debated issue. Ozkan and Erden (2015), Carrière-Swallow et al. (2021), and Gust et al. (2010) document a downward trend, while Bouakez and Rebei (2008) find that the effects of exchange rates on import prices have been stable since the 1990s. In addition, several studies suggest that there has been a revival in exchange rate pass-through since the financial crisis (Benigno & Faia, 2016; Shioji, 2015; Ozkan & Erden, 2015). Figure 1 shows the time varying pass-through along with 90intervals. There is little evidence of a negative trend as only two of the eight countries show a distinctly declining pass-through since 1985 (Australia and Japan). While Shioji (2015) conclude that the Japanese pass-through accelerated after 2015, our estimates merely indicate that the downward trend was discontinued around this time.

### Pass-through and macroeconomic variables

How much an importing firm adjust its domestic currency price as the nominal exchange rate changes depends on the time series properties of the variables involved. Devereux and Yetman (2010) develop a model with Calvo pricing and find that the pass-through of exchange rate changes is positively related to the levels and variances of the exchange rate and domestic inflation. Kasa (1992) models pricing to market or incomplete pass-through of real exchange rate changes where price adjustment is costly. Pass-through increases with the persistence of the nominal exchange rate because is not optimal for the firm to pay the adjustment cost if it is likely that the change is temporary. In the Taylor (2000) model, firms with market power set prices for several periods, but do not face adjustment costs. More persistent movements in exchange rates, foreign prices, and domestic inflation lead to higher pass-through in this model as well. When the inflation target is more credible, the persistence of shocks to inflation is lower, and it is optimal for the firm to change its price less in response to a given exchange rate shock. Hence the level, variance, and persistence of inflation and exchange rates should affect pass-through. Several models also imply that aggregate demand is important, with higher pass-through during favorable conditions (Kasa, 1992; Menon, 1995).

Empirically, the level of domestic inflation has received the most attention in this context, with a well-documented positive relationship to pass-through (Campa & Goldberg, 2005; Choudhri & Hakura, 2006; Devereux & Yetman, 2010). Similarly, inflation volatility is included in a number of studies. Campa and Goldberg (2005) and Devereux and Yetman (2010) document positive relationships between pass-through and exchange rate volatility, while Ozkan and Erden (2015) find a negative effect. Contrary to theory, the latter also observe a negative association to aggregate demand as measured by the output gap. The persistence of shocks to inflation and the exchange rate is important in theoretical models, but has received less attention in empirical studies. An and Wang (2012) and McCarthy (2007) document a positive association between exchange rate persistence and pass-through using time invariant correlations in cross country data.

Table 1 contains the results from regressing time varying pass-through on a set of variables. Standard errors are clustered by time and country.^1^ Several subsamples are studied: The inflation targeting period 1993–2021, observations with inflation below 1observations with exchange rate appreciations.

**Table 1:** Time varying pass through and macro variables

	1985–2021	1993–2021	$\Delta CPI<0.01$	$\Delta E<0$	1985–2021
$$\Delta CPI$$	$$0.00132^{**}$$	0.00108	0.00135	0.00093	
	2.331	0.670	1.568	1.344	
*Var*(*CPI*)	$$0.0901^{***}$$	$$0.0363^{**}$$	$$0.0813^{***}$$	$$0.0823^{***}$$	
	3.565	2.261	3.452	3.226	
*AR*(*CPI*)	$$0.00617^{***}$$	$$0.00164^{***}$$	$$0.00952^{***}$$	$$0.0119^{***}$$	
	3.782	2.810	3.145	3.696	
$$\Delta E$$	$$0.342^{***}$$	$$0.657^{***}$$	0.260	$$0.564^{**}$$	$$0.598^{***}$$
	4.043	5.082	1.212	2.414	6.624
*Var*(*E*)	0.531	$$0.622^{***}$$	$$0.623^{**}$$	0.469	$$0.399^{***}$$
	1.358	3.011	1.968	1.530	4.307
*AR*(*E*)	0.492	0.495	$$0.382^{**}$$	$$0.625^{**}$$	0.301
	0.746	1.012	2.182	1.923	0.362
*Y*	$$0.0213^{*}$$	$$0.0466^{**}$$	0.0204	0.0641	
	1.702	2.571	1.582	1.404	
*Open*	$$-0.00245^{***}$$	$$-0.00209^{***}$$	$$-0.00197^{***}$$	$$-0.00164^{***}$$	
	2.598	2.255	2.018	1.716	
# Obs	1176	928	424	452	1176

Dependent variable is the response of domestic prices to exchange rate shocks after four quarters. *t*-statistics within parenthesis. *AR(x)* is the persistence of *x*. Country fixed effects are included. Standard errors are clustered by time and country

***, **, and * denote, respectively, significance at the 1%, 5%, and 10% levels

Consistent with previous studies, we find that exchange rate pass-through increases with domestic inflation for the full sample as well as for the inflation targeting period. However, this relationship disappears when observations with low inflation are isolated. Higher inflation variance is also associated with higher exchange rate pass-through, which is consistent with the model of Devereux and Yetman (2010) as well as with previous studies (Ozkan & Erden, 2015). Previous results are mixed concerning the volatility of exchange rates as Campa and Goldberg (2005) find a positive association and Ozkan and Erden (2015) a negative. For our data, the volatility of exchange rate shocks has a significant relationship to pass-through only for the sample 1993–2021 and observations with low inflation.

Higher persistence of exchange rate shocks increases pass-through in theory if prices are sticky because of adjustment costs or time dependent pricing rules. This relationship is significant for two subsamples: Observations with low inflation and observations with exchange rate appreciations. Consistent with theory and previous studies, pass-through consistently has a negative relationship to openness and a positive relationship to the change of the exchange rate. The latter finding implies that depreciations are passed through to domestic prices to a higher degree than appreciations, which makes sense given that prices are increased regularly as long as the inflation rate is positive.

Aggregate demand as measured by the output gap enters with positive coefficients throughout Table 1 and the relationship is significant for the for the full sample as well as for the 1993–2021 period. Previous empirical evidence is mixed as Brun-Aguerre et al. (2012) documents a negative relationship between the output gap and pass-through, while Delatte and López-Villavicencio (2012) have different results for different currency pairs. The final column shows that the results are qualitatively unchanged when only the properties of the nominal exchange rate are included.

## Conclusions

We estimate the pass-through of nominal exchange rate shocks to domestic prices using Bayesian VAR models with time-varying parameters and stochastic volatility. The time varying pass-through coefficients are then regressed on a set of macroeconomic variables. Allowing continuous time variation provides more information on the behavior of pass-through than cross country regressions and/or split sample estimation, which is used in most previous studies.

The level of inflation displays a positive effect for the full sample, but not for any of the subsamples. Instead, we systematically find positive relationships between exchange rate pass-through and the variance and persistence of shocks to domestic prices. Exchange rate persistence is associated high pass-through only for observations with exchange rate appreciations or low inflation. The effect of persistence of inflation is also much stronger for these two subsamples. Aggregate demand has a significant positive relationship to pass-through for the full sample as well as for the 1993–2021 period. Overall, we find that the pass-through of exchange rates to import prices varies considerably over time in a manner which is consistent with microeconomic pricing models and macroeconomic theory.

## Footnotes

1 For country* i* and time *t*, we allow $Var\left( \varepsilon _{it}\right) =\sigma _{it}$, and $Cov\left( \varepsilon _{it},\varepsilon _{is}\right) =\sigma _{is},$ but the covariance terms $Cov\left( \varepsilon _{it},\varepsilon _{is}\right) =\sigma _{ts}$ are assumed to be constant over time.
